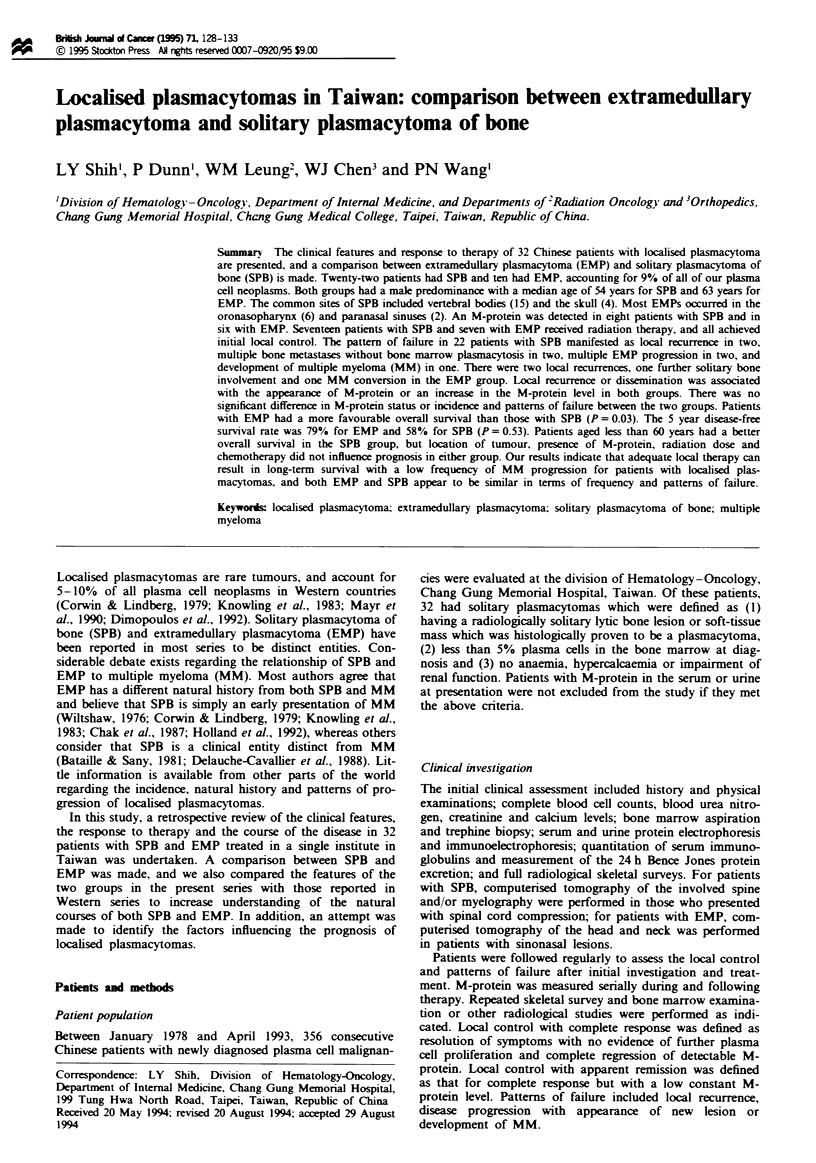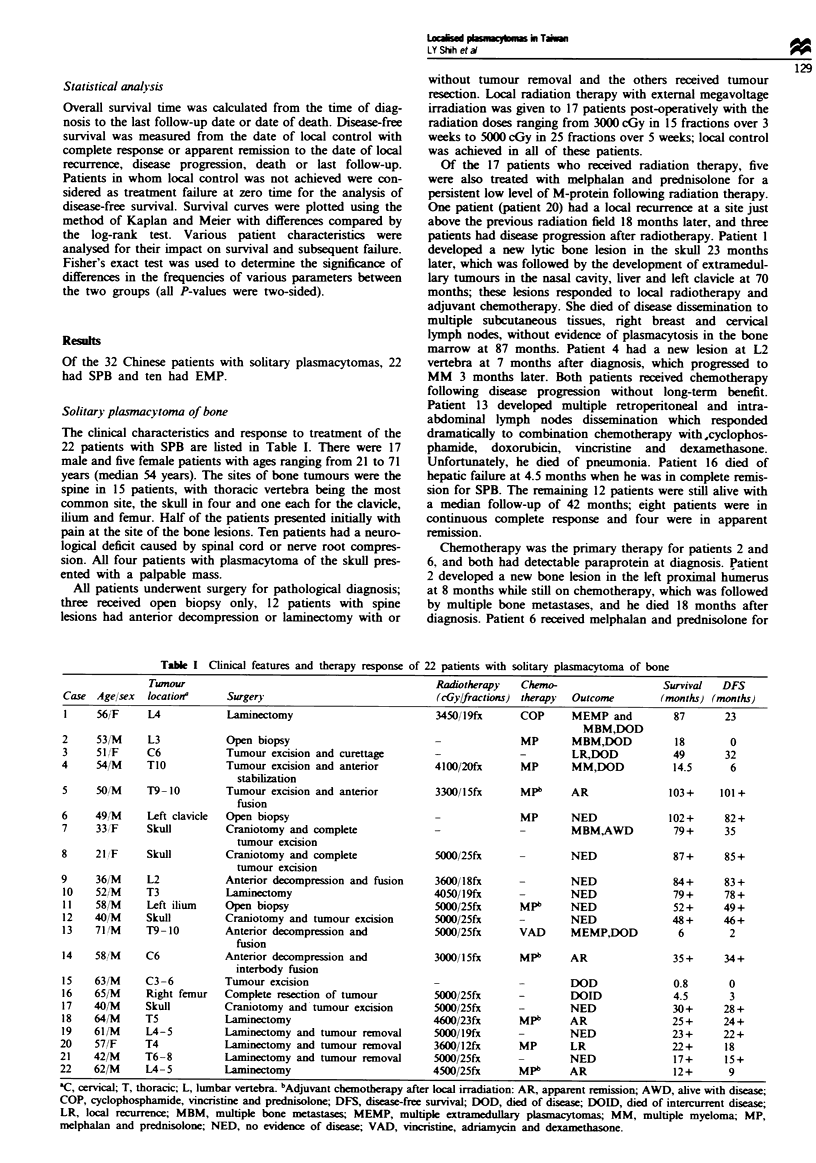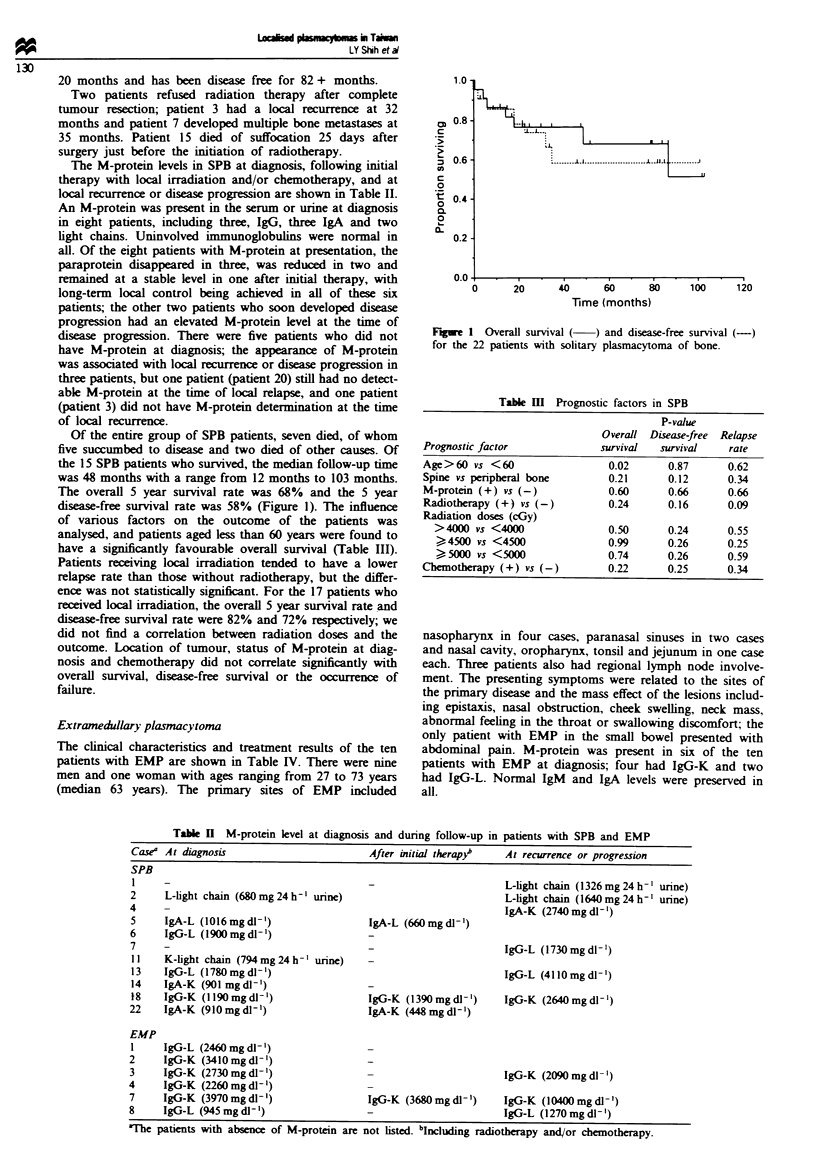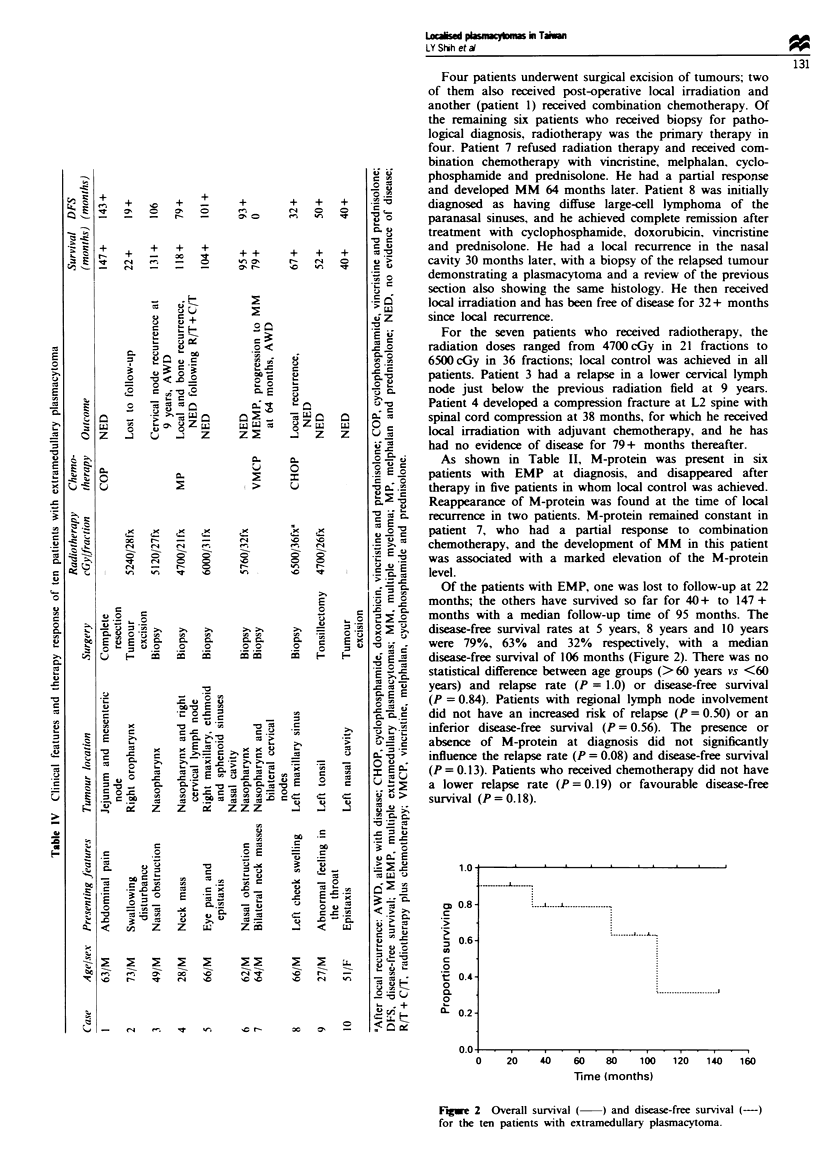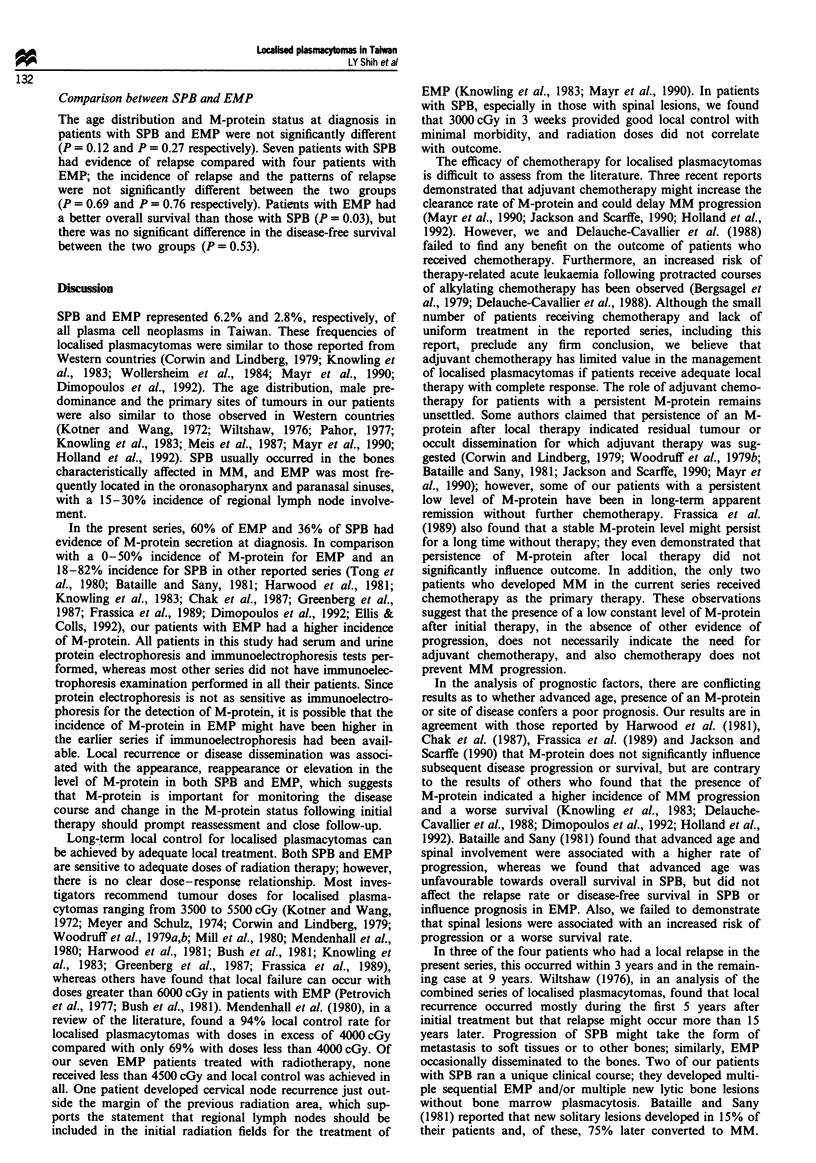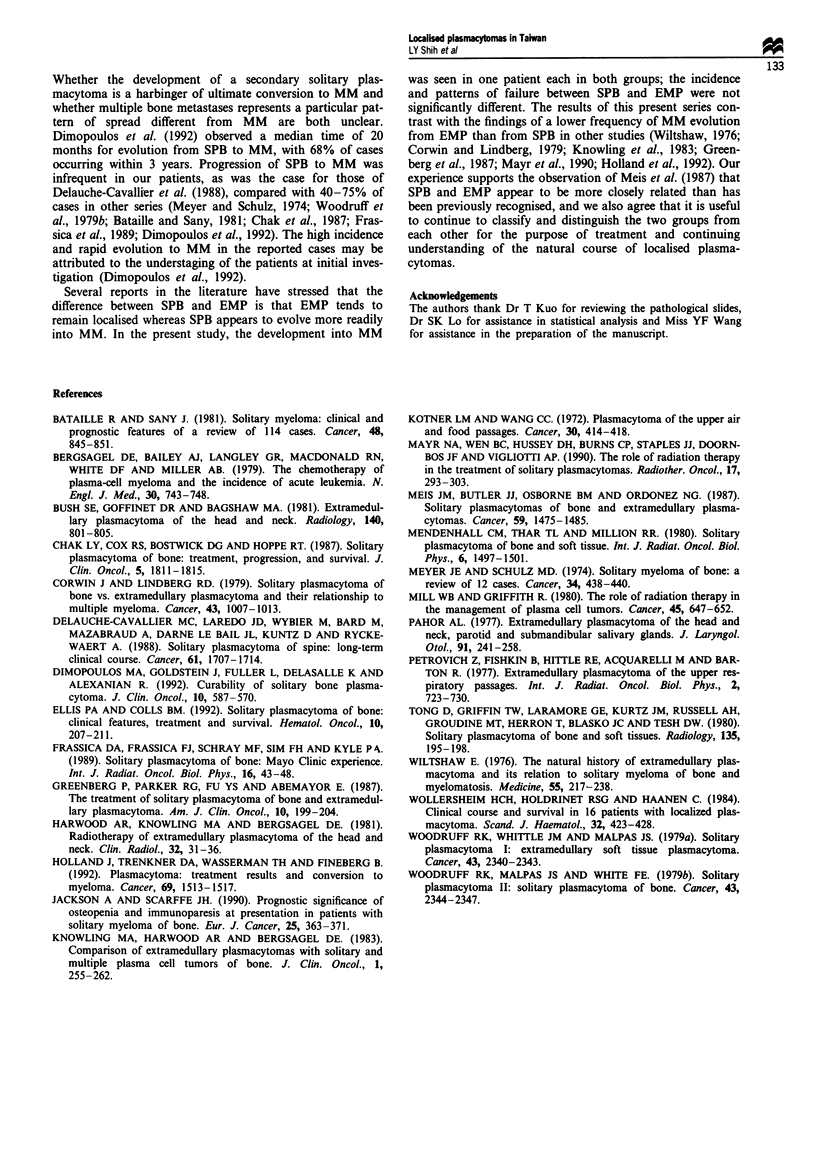# Localised plasmacytomas in Taiwan: comparison between extramedullary plasmacytoma and solitary plasmacytoma of bone.

**DOI:** 10.1038/bjc.1995.26

**Published:** 1995-01

**Authors:** L. Y. Shih, P. Dunn, W. M. Leung, W. J. Chen, P. N. Wang

**Affiliations:** Department of Internal Medicine, Chang Gung Memorial Hospital, Chang Gung Medical College, Taipei, Taiwan, Republic of China.

## Abstract

The clinical features and response to therapy of 32 Chinese patients with localised plasmacytoma are presented, and a comparison between extramedullary plasmacytoma (EMP) and solitary plasmacytoma of bone (SPB) is made. Twenty-two patients had SPB and ten had EMP, accounting for 9% of all of our plasma cell neoplasms. Both groups had a male predominance with a median age of 54 years for SPB and 63 years for EMP. The common sites of SPB included vertebral bodies (15) and the skull (4). Most EMPs occurred in the oronasopharynx (6) and paranasal sinuses (2). An M-protein was detected in eight patients with SPB and in six with EMP. Seventeen patients with SPB and seven with EMP received radiation therapy, and all achieved initial local control. The pattern of failure in 22 patients with SPB manifested as local recurrence in two, multiple bone metastases without bone marrow plasmacytosis in two, multiple EMP progression in two, and development of multiple myeloma (MM) in one. There were two local recurrences, one further solitary bone involvement and one MM conversion in the EMP group. Local recurrence or dissemination was associated with the appearance of M-protein or an increase in the M-protein level in both groups. There was no significant difference in M-protein status or incidence and patterns of failure between the two groups. Patients with EMP had a more favourable overall survival than those with SPB (P = 0.03). The 5 year disease-free survival rate was 79% for EMP and 58% for SPB (P = 0.53). Patients aged less than 60 years had a better overall survival in the SPB group, but location of tumour, presence of M-protein, radiation dose and chemotherapy did not influence prognosis in either group. Our results indicate that adequate local therapy can result in long-term survival with a low frequency of MM progression for patients with localised plasmacytomas, and both EMP and SPB appear to be similar in terms of frequency and patterns of failure.


British Journal of Cancer (1995) 71, 128-133

© 1995 Stockton Press All rights reserved 0007-0920/95 $9.00

### Localised plasmacytomas in Taiwan: comparison between extramedullary plasmacytoma and solitary plasmacytoma of bone

LY Shih[1], P Dunn[1], WM Leung[2], WJ Chen[3] and PN Wang[1]

[1]*Division of Hematology-Oncology, Department of Internal Medicine, and Departments of* [2]*Radiation Oncology and* [3]*Orthopedics, Chang Gung Memorial Hospital, Chang Gung Medical College, Taipei, Taiwan, Republic of China.*

**Summary** The clinical features and response to therapy of 32 Chinese patients with localised plasmacytoma are presented, and a comparison between extramedullary plasmacytoma (EMP) and solitary plasmacytoma of bone (SPB) is made. Twenty-two patients had SPB and ten had EMP, accounting for 9% of all of our plasma cell neoplasms. Both groups had a male predominance with a median age of 54 years for SPB and 63 years for EMP. The common sites of SPB included vertebral bodies (15) and the skull (4). Most EMPs occurred in the oronasopharynx (6) and paranasal sinuses (2). An M-protein was detected in eight patients with SPB and in six with EMP. Seventeen patients with SPB and seven with EMP received radiation therapy, and all achieved initial local control. The pattern of failure in 22 patients with SPB manifested as local recurrence in two, multiple bone metastases without bone marrow plasmacytosis in two, multiple EMP progression in two, and development of multiple myeloma (MM) in one. There were two local recurrences, one further solitary bone involvement and one MM conversion in the EMP group. Local recurrence or dissemination was associated with the appearance of M-protein or an increase in the M-protein level in both groups. There was no significant difference in M-protein status or incidence and patterns of failure between the two groups. Patients with EMP had a more favourable overall survival than those with SPB ($P = 0.03$). The 5 year disease-free survival rate was 79% for EMP and 58% for SPB ($P = 0.53$). Patients aged less than 60 years had a better overall survival in the SPB group, but location of tumour, presence of M-protein, radiation dose and chemotherapy did not influence prognosis in either group. Our results indicate that adequate local therapy can result in long-term survival with a low frequency of MM progression for patients with localised plasmacytomas, and both EMP and SPB appear to be similar in terms of frequency and patterns of failure.

**Keywords:** localised plasmacytoma; extramedullary plasmacytoma; solitary plasmacytoma of bone; multiple myeloma

Localised plasmacytomas are rare tumours, and account for 5-10% of all plasma cell neoplasms in Western countries (Corwin & Lindberg, 1979; Knowling *et al.*, 1983; Mayr *et al.*, 1990; Dimopoulos *et al.*, 1992). Solitary plasmacytoma of bone (SPB) and extramedullary plasmacytoma (EMP) have been reported in most series to be distinct entities. Considerable debate exists regarding the relationship of SPB and EMP to multiple myeloma (MM). Most authors agree that EMP has a different natural history from both SPB and MM and believe that SPB is simply an early presentation of MM (Wiltshaw, 1976; Corwin & Lindberg, 1979; Knowling *et al.*, 1983; Chak *et al.*, 1987; Holland *et al.*, 1992), whereas others consider that SPB is a clinical entity distinct from MM (Bataille & Sany, 1981; Delauche-Cavallier *et al.*, 1988). Little information is available from other parts of the world regarding the incidence, natural history and patterns of progression of localised plasmacytomas.

In this study, a retrospective review of the clinical features, the response to therapy and the course of the disease in 32 patients with SPB and EMP treated in a single institute in Taiwan was undertaken. A comparison between SPB and EMP was made, and we also compared the features of the two groups in the present series with those reported in Western series to increase understanding of the natural courses of both SPB and EMP. In addition, an attempt was made to identify the factors influencing the prognosis of localised plasmacytomas.

### Patients and methods

### *Patient population*

Between January 1978 and April 1993, 356 consecutive Chinese patients with newly diagnosed plasma cell malignancies were evaluated at the division of Hematology-Oncology, Chang Gung Memorial Hospital, Taiwan. Of these patients, 32 had solitary plasmacytomas which were defined as (1) having a radiologically solitary lytic bone lesion or soft-tissue mass which was histologically proven to be a plasmacytoma, (2) less than 5% plasma cells in the bone marrow at diagnosis and (3) no anaemia, hypercalcaemia or impairment of renal function. Patients with M-protein in the serum or urine at presentation were not excluded from the study if they met the above criteria.

### *Clinical investigation*

The initial clinical assessment included history and physical examinations; complete blood cell counts, blood urea nitrogen, creatinine and calcium levels; bone marrow aspiration and trephine biopsy; serum and urine protein electrophoresis and immunoelectrophoresis; quantitation of serum immunoglobulins and measurement of the 24 h Bence Jones protein excretion; and full radiological skeletal surveys. For patients with SPB, computerised tomography of the involved spine and/or myelography were performed in those who presented with spinal cord compression; for patients with EMP, computerised tomography of the head and neck was performed in patients with sinonasal lesions.

Patients were followed regularly to assess the local control and patterns of failure after initial investigation and treatment. M-protein was measured serially during and following therapy. Repeated skeletal survey and bone marrow examination or other radiological studies were performed as indicated. Local control with complete response was defined as resolution of symptoms with no evidence of further plasma cell proliferation and complete regression of detectable M-protein. Local control with apparent remission was defined as that for complete response but with a low constant M-protein level. Patterns of failure included local recurrence, disease progression with appearance of new lesion or development of MM.

Correspondence: LY Shih, Division of Hematology-Oncology, Department of Internal Medicine, Chang Gung Memorial Hospital, 199 Tung Hwa North Road, Taipei, Taiwan, Republic of China
Received 20 May 1994; revised 20 August 1994; accepted 29 August 1994

*Statistical analysis*

Overall survival time was calculated from the time of diagnosis to the last follow-up date or date of death. Disease-free survival was measured from the date of local control with complete response or apparent remission to the date of local recurrence, disease progression, death or last follow-up. Patients in whom local control was not achieved were considered as treatment failure at zero time for the analysis of disease-free survival. Survival curves were plotted using the method of Kaplan and Meier with differences compared by the log-rank test. Various patient characteristics were analysed for their impact on survival and subsequent failure. Fisher's exact test was used to determine the significance of differences in the frequencies of various parameters between the two groups (all *P*-values were two-sided).

### Results

Of the 32 Chinese patients with solitary plasmacytomas, 22 had SPB and ten had EMP.

*Solitary plasmacytoma of bone*

The clinical characteristics and response to treatment of the 22 patients with SPB are listed in Table I. There were 17 male and five female patients with ages ranging from 21 to 71 years (median 54 years). The sites of bone tumours were the spine in 15 patients, with thoracic vertebra being the most common site, the skull in four and one each for the clavicle, ilium and femur. Half of the patients presented initially with pain at the site of the bone lesions. Ten patients had a neurological deficit caused by spinal cord or nerve root compression. All four patients with plasmacytoma of the skull presented with a palpable mass.

All patients underwent surgery for pathological diagnosis; three received open biopsy only, 12 patients with spine lesions had anterior decompression or laminectomy with or without tumour removal and the others received tumour resection. Local radiation therapy with external megavoltage irradiation was given to 17 patients post-operatively with the radiation doses ranging from 3000 cGy in 15 fractions over 3 weeks to 5000 cGy in 25 fractions over 5 weeks; local control was achieved in all of these patients.

Of the 17 patients who received radiation therapy, five were also treated with melphalan and prednisolone for a persistent low level of M-protein following radiation therapy. One patient (patient 20) had a local recurrence at a site just above the previous radiation field 18 months later, and three patients had disease progression after radiotherapy. Patient 1 developed a new lytic bone lesion in the skull 23 months later, which was followed by the development of extramedullary tumours in the nasal cavity, liver and left clavicle at 70 months; these lesions responded to local radiotherapy and adjuvant chemotherapy. She died of disease dissemination to multiple subcutaneous tissues, right breast and cervical lymph nodes, without evidence of plasmacytosis in the bone marrow at 87 months. Patient 4 had a new lesion at L2 vertebra at 7 months after diagnosis, which progressed to MM 3 months later. Both patients received chemotherapy following disease progression without long-term benefit. Patient 13 developed multiple retroperitoneal and intra-abdominal lymph nodes dissemination which responded dramatically to combination chemotherapy with cyclophosphamide, doxorubicin, vincristine and dexamethasone. Unfortunately, he died of pneumonia. Patient 16 died of hepatic failure at 4.5 months when he was in complete remission for SPB. The remaining 12 patients were still alive with a median follow-up of 42 months; eight patients were in continuous complete response and four were in apparent remission.

Chemotherapy was the primary therapy for patients 2 and 6, and both had detectable paraprotein at diagnosis. Patient 2 developed a new bone lesion in the left proximal humerus at 8 months while still on chemotherapy, which was followed by multiple bone metastases, and he died 18 months after diagnosis. Patient 6 received melphalan and prednisolone for

**Table I** Clinical features and therapy response of 22 patients with solitary plasmacytoma of bone

| *Case* | *Age/sex* | *Tumour location*[a] | *Surgery* | *Radiotherapy (cGy/fractions)* | *Chemo-therapy* | *Outcome* | *Survival (months)* | *DFS (months)* |
|---|---|---|---|---|---|---|---|---|
| 1 | 56/F | L4 | Laminectomy | 3450/19fx | COP | MEMP and MBM,DOD | 87 | 23 |
| 2 | 53/M | L3 | Open biopsy | – | MP | MBM,DOD | 18 | 0 |
| 3 | 51/F | C6 | Tumour excision and curettage | – | – | LR,DOD | 49 | 32 |
| 4 | 54/M | T10 | Tumour excision and anterior stabilization | 4100/20fx | MP | MM,DOD | 14.5 | 6 |
| 5 | 50/M | T9–10 | Tumour excision and anterior fusion | 3300/15fx | MP[b] | AR | 103+ | 101+ |
| 6 | 49/M | Left clavicle | Open biopsy | – | MP | NED | 102+ | 82+ |
| 7 | 33/F | Skull | Craniotomy and complete tumour excision | – | – | MBM,AWD | 79+ | 35 |
| 8 | 21/F | Skull | Craniotomy and complete tumour excision | 5000/25fx | – | NED | 87+ | 85+ |
| 9 | 36/M | L2 | Anterior decompression and fusion | 3600/18fx | – | NED | 84+ | 83+ |
| 10 | 52/M | T3 | Laminectomy | 4050/19fx | – | NED | 79+ | 78+ |
| 11 | 58/M | Left ilium | Open biopsy | 5000/25fx | MP[b] | NED | 52+ | 49+ |
| 12 | 40/M | Skull | Craniotomy and tumour excision | 5000/25fx | – | NED | 48+ | 46+ |
| 13 | 71/M | T9–10 | Anterior decompression and fusion | 5000/25fx | VAD | MEMP,DOD | 6 | 2 |
| 14 | 58/M | C6 | Anterior decompression and interbody fusion | 3000/15fx | MP[b] | AR | 35+ | 34+ |
| 15 | 63/M | C3–6 | Tumour excision | – | – | DOD | 0.8 | 0 |
| 16 | 65/M | Right femur | Complete resection of tumour | 5000/25fx | – | DOID | 4.5 | 3 |
| 17 | 40/M | Skull | Craniotomy and tumour excision | 5000/25fx | – | NED | 30+ | 28+ |
| 18 | 64/M | T5 | Laminectomy | 4600/23fx | MP[b] | AR | 25+ | 24+ |
| 19 | 61/M | L4–5 | Laminectomy and tumour removal | 5000/19fx | – | NED | 23+ | 22+ |
| 20 | 57/F | T4 | Laminectomy and tumour removal | 3600/12fx | MP | LR | 22+ | 18 |
| 21 | 42/M | T6–8 | Laminectomy and tumour removal | 5000/25fx | – | NED | 17+ | 15+ |
| 22 | 62/M | L4–5 | Laminectomy | 4500/25fx | MP[b] | AR | 12+ | 9 |

[a]C, cervical; T, thoracic; L, lumbar vertebra. [b]Adjuvant chemotherapy after local irradiation: AR, apparent remission; AWD, alive with disease; COP, cyclophosphamide, vincristine and prednisolone; DFS, disease-free survival; DOD, died of disease; DOID, died of intercurrent disease; LR, local recurrence; MBM, multiple bone metastases; MEMP, multiple extramedullary plasmacytomas; MM, multiple myeloma; MP, melphalan and prednisolone; NED, no evidence of disease; VAD, vincristine, adriamycin and dexamethasone.

20 months and has been disease free for 82+ months.

Two patients refused radiation therapy after complete tumour resection; patient 3 had a local recurrence at 32 months and patient 7 developed multiple bone metastases at 35 months. Patient 15 died of suffocation 25 days after surgery just before the initiation of radiotherapy.

The M-protein levels in SPB at diagnosis, following initial therapy with local irradiation and/or chemotherapy, and at local recurrence or disease progression are shown in Table II. An M-protein was present in the serum or urine at diagnosis in eight patients, including three, IgG, three IgA and two light chains. Uninvolved immunoglobulins were normal in all. Of the eight patients with M-protein at presentation, the paraprotein disappeared in three, was reduced in two and remained at a stable level in one after initial therapy, with long-term local control being achieved in all of these six patients; the other two patients who soon developed disease progression had an elevated M-protein level at the time of disease progression. There were five patients who did not have M-protein at diagnosis; the appearance of M-protein was associated with local recurrence or disease progression in three patients, but one patient (patient 20) still had no detectable M-protein at the time of local relapse, and one patient (patient 3) did not have M-protein determination at the time of local recurrence.

Of the entire group of SPB patients, seven died, of whom five succumbed to disease and two died of other causes. Of the 15 SPB patients who survived, the median follow-up time was 48 months with a range from 12 months to 103 months. The overall 5 year survival rate was 68% and the 5 year disease-free survival rate was 58% (Figure 1). The influence of various factors on the outcome of the patients was analysed, and patients aged less than 60 years were found to have a significantly favourable overall survival (Table III). Patients receiving local irradiation tended to have a lower relapse rate than those without radiotherapy, but the difference was not statistically significant. For the 17 patients who received local irradiation, the overall 5 year survival rate and disease-free survival rate were 82% and 72% respectively; we did not find a correlation between radiation doses and the outcome. Location of tumour, status of M-protein at diagnosis and chemotherapy did not correlate significantly with overall survival, disease-free survival or the occurrence of failure.

**Figure 1** Overall survival (——) and disease-free survival (----) for the 22 patients with solitary plasmacytoma of bone.

**Table III** Prognostic factors in SPB

| *Prognostic factor* | *Overall survival* (P-*value*) | *Disease-free survival* (P-*value*) | *Relapse rate* (P-*value*) |
|---|---|---|---|
| Age>60 *vs* <60 | 0.02 | 0.87 | 0.62 |
| Spine *vs* peripheral bone | 0.21 | 0.12 | 0.34 |
| M-protein (+) *vs* (−) | 0.60 | 0.66 | 0.66 |
| Radiotherapy (+) *vs* (−) | 0.24 | 0.16 | 0.09 |
| Radiation doses (cGy) | | | |
| >4000 *vs* <4000 | 0.50 | 0.24 | 0.55 |
| ⩾4500 *vs* <4500 | 0.99 | 0.26 | 0.25 |
| ⩾5000 *vs* <5000 | 0.74 | 0.26 | 0.59 |
| Chemotherapy (+) *vs* (−) | 0.22 | 0.25 | 0.34 |

### *Extramedullary plasmacytoma*

The clinical characteristics and treatment results of the ten patients with EMP are shown in Table IV. There were nine men and one woman with ages ranging from 27 to 73 years (median 63 years). The primary sites of EMP included nasopharynx in four cases, paranasal sinuses in two cases and nasal cavity, oropharynx, tonsil and jejunum in one case each. Three patients also had regional lymph node involvement. The presenting symptoms were related to the sites of the primary disease and the mass effect of the lesions including epistaxis, nasal obstruction, cheek swelling, neck mass, abnormal feeling in the throat or swallowing discomfort; the only patient with EMP in the small bowel presented with abdominal pain. M-protein was present in six of the ten patients with EMP at diagnosis; four had IgG-K and two had IgG-L. Normal IgM and IgA levels were preserved in all.

**Table II** M-protein level at diagnosis and during follow-up in patients with SPB and EMP

| *Case*[a] | *At diagnosis* | *After initial therapy*[b] | *At recurrence or progression* |
|---|---|---|---|
| *SPB* | | | |
| 1 | – | – | L-light chain ($1326\ mg\ 24\ h^{-1}$ urine) |
| 2 | L-light chain ($680\ mg\ 24\ h^{-1}$ urine) | | L-light chain ($1640\ mg\ 24\ h^{-1}$ urine) |
| 4 | – | | IgA-K ($2740\ mg\ dl^{-1}$) |
| 5 | IgA-L ($1016\ mg\ dl^{-1}$) | IgA-L ($660\ mg\ dl^{-1}$) | |
| 6 | IgG-L ($1900\ mg\ dl^{-1}$) | – | |
| 7 | – | – | IgG-L ($1730\ mg\ dl^{-1}$) |
| 11 | K-light chain ($794\ mg\ 24\ h^{-1}$ urine) | – | |
| 13 | IgG-L ($1780\ mg\ dl^{-1}$) | | IgG-L ($4110\ mg\ dl^{-1}$) |
| 14 | IgA-K ($901\ mg\ dl^{-1}$) | – | |
| 18 | IgG-K ($1190\ mg\ dl^{-1}$) | IgG-K ($1390\ mg\ dl^{-1}$) | IgG-K ($2640\ mg\ dl^{-1}$) |
| 22 | IgA-K ($910\ mg\ dl^{-1}$) | IgA-K ($448\ mg\ dl^{-1}$) | |
| *EMP* | | | |
| 1 | IgG-L ($2460\ mg\ dl^{-1}$) | – | |
| 2 | IgG-K ($3410\ mg\ dl^{-1}$) | – | |
| 3 | IgG-K ($2730\ mg\ dl^{-1}$) | – | IgG-K ($2090\ mg\ dl^{-1}$) |
| 4 | IgG-K ($2260\ mg\ dl^{-1}$) | – | |
| 7 | IgG-K ($3970\ mg\ dl^{-1}$) | IgG-K ($3680\ mg\ dl^{-1}$) | IgG-K ($10400\ mg\ dl^{-1}$) |
| 8 | IgG-L ($945\ mg\ dl^{-1}$) | – | IgG-L ($1270\ mg\ dl^{-1}$) |

[a]The patients with absence of M-protein are not listed. [b]Including radiotherapy and/or chemotherapy.

**Table IV** Clinical features and therapy response of ten patients with extramedullary plasmacytoma

| *Case* | *Age/sex* | *Presenting features* | *Tumour location* | *Surgery* | *Radiotherapy cGy/fraction* | *Chemo-therapy* | *Outcome* | *Survival (months)* | *DFS (months)* |
|---|---|---|---|---|---|---|---|---|---|
| 1 | 63/M | Abdominal pain | Jejunum and mesenteric node | Complete resection | – | COP | NED | 147+ | 143+ |
| 2 | 73/M | Swallowing disturbance | Right oropharynx | Tumour excision | 5240/28fx | | Lost to follow-up | 22+ | 19+ |
| 3 | 49/M | Nasal obstruction | Nasopharynx | Biopsy | 5120/27fx | | Cervical node recurrence at 9 years, AWD | 131+ | 106 |
| 4 | 28/M | Neck mass | Nasopharynx and right cervical lymph node | Biopsy | 4700/21fx | MP | Local and bone recurrence, NED following R/T+C/T | 118+ | 79+ |
| 5 | 66/M | Eye pain and epistaxis | Right maxillary, ethmoid and sphenoid sinuses Nasal cavity | Biopsy | 6000/31fx | | NED | 104+ | 101+ |
| 6 | 62/M | Nasal obstruction | Nasopharynx | Biopsy | 5760/32fx | – | NED | 95+ | 93+ |
| 7 | 64/M | Bilateral neck masses | Nasopharynx and bilateral cervical nodes | Biopsy | | VMCP | MEMP, progression to MM at 64 months, AWD | 79+ | 0 |
| 8 | 66/M | Left cheek swelling | Left maxillary sinus | Biopsy | 6500/36fx[a] | CHOP | Local recurrence, NED | 67+ | 32+ |
| 9 | 27/M | Abnormal feeling in the throat | Left tonsil | Tonsillectomy | 4700/26fx | | NED | 52+ | 50+ |
| 10 | 51/F | Epistaxis | Left nasal cavity | Tumour excision | – | | NED | 40+ | 40+ |

[a]After local recurrence: AWD, alive with disease; CHOP, cyclophosphamide, doxorubicin, vincristine and prednisolone; COP, cyclophosphamide, vincristine and prednisolone; DFS, disease-free survival; MEMP, multiple extramedullary plasmacytomas; MM, multiple myeloma; MP, melphalan and prednisolone; NED, no evidence of disease; R/T + C/T, radiotherapy plus chemotherapy; VMCP, vincristine, melphalan, cyclophosphamide and prednisolone.

Four patients underwent surgical excision of tumours; two of them also received post-operative local irradiation and another (patient 1) received combination chemotherapy. Of the remaining six patients who received biopsy for pathological diagnosis, radiotherapy was the primary therapy in four. Patient 7 refused radiation therapy and received combination chemotherapy with vincristine, melphalan, cyclophosphamide and prednisolone. He had a partial response and developed MM 64 months later. Patient 8 was initially diagnosed as having diffuse large-cell lymphoma of the paranasal sinuses, and he achieved complete remission after treatment with cyclophosphamide, doxorubicin, vincristine and prednisolone. He had a local recurrence in the nasal cavity 30 months later, with a biopsy of the relapsed tumour demonstrating a plasmacytoma and a review of the previous section also showing the same histology. He then received local irradiation and has been free of disease for 32+ months since local recurrence.

For the seven patients who received radiotherapy, the radiation doses ranged from 4700 cGy in 21 fractions to 6500 cGy in 36 fractions; local control was achieved in all patients. Patient 3 had a relapse in a lower cervical lymph node just below the previous radiation field at 9 years. Patient 4 developed a compression fracture at L2 spine with spinal cord compression at 38 months, for which he received local irradiation with adjuvant chemotherapy, and he has had no evidence of disease for 79+ months thereafter.

As shown in Table II, M-protein was present in six patients with EMP at diagnosis, and disappeared after therapy in five patients in whom local control was achieved. Reappearance of M-protein was found at the time of local recurrence in two patients. M-protein remained constant in patient 7, who had a partial response to combination chemotherapy, and the development of MM in this patient was associated with a marked elevation of the M-protein level.

Of the patients with EMP, one was lost to follow-up at 22 months; the others have survived so far for 40+ to 147+ months with a median follow-up time of 95 months. The disease-free survival rates at 5 years, 8 years and 10 years were 79%, 63% and 32% respectively, with a median disease-free survival of 106 months (Figure 2). There was no statistical difference between age groups (>60 years *vs* <60 years) and relapse rate ($P = 1.0$) or disease-free survival ($P = 0.84$). Patients with regional lymph node involvement did not have an increased risk of relapse ($P = 0.50$) or an inferior disease-free survival ($P = 0.56$). The presence or absence of M-protein at diagnosis did not significantly influence the relapse rate ($P = 0.08$) and disease-free survival ($P = 0.13$). Patients who received chemotherapy did not have a lower relapse rate ($P = 0.19$) or favourable disease-free survival ($P = 0.18$).

**Figure 2** Overall survival (——) and disease-free survival (----) for the ten patients with extramedullary plasmacytoma.

*Comparison between SPB and EMP*

The age distribution and M-protein status at diagnosis in patients with SPB and EMP were not significantly different ($P = 0.12$ and $P = 0.27$ respectively). Seven patients with SPB had evidence of relapse compared with four patients with EMP; the incidence of relapse and the patterns of relapse were not significantly different between the two groups ($P = 0.69$ and $P = 0.76$ respectively). Patients with EMP had a better overall survival than those with SPB ($P = 0.03$), but there was no significant difference in the disease-free survival between the two groups ($P = 0.53$).

### Discussion

SPB and EMP represented 6.2% and 2.8%, respectively, of all plasma cell neoplasms in Taiwan. These frequencies of localised plasmacytomas were similar to those reported from Western countries (Corwin and Lindberg, 1979; Knowling *et al*., 1983; Wollersheim *et al*., 1984; Mayr *et al*., 1990; Dimopoulos *et al*., 1992). The age distribution, male predominance and the primary sites of tumours in our patients were also similar to those observed in Western countries (Kotner and Wang, 1972; Wiltshaw, 1976; Pahor, 1977; Knowling *et al*., 1983; Meis *et al*., 1987; Mayr *et al*., 1990; Holland *et al*., 1992). SPB usually occurred in the bones characteristically affected in MM, and EMP was most frequently located in the oronasopharynx and paranasal sinuses, with a 15–30% incidence of regional lymph node involvement.

In the present series, 60% of EMP and 36% of SPB had evidence of M-protein secretion at diagnosis. In comparison with a 0–50% incidence of M-protein for EMP and an 18–82% incidence for SPB in other reported series (Tong *et al*., 1980; Bataille and Sany, 1981; Harwood *et al*., 1981; Knowling *et al*., 1983; Chak *et al*., 1987; Greenberg *et al*., 1987; Frassica *et al*., 1989; Dimopoulos *et al*., 1992; Ellis & Colls, 1992), our patients with EMP had a higher incidence of M-protein. All patients in this study had serum and urine protein electrophoresis and immunoelectrophoresis tests performed, whereas most other series did not have immunoelectrophoresis examination performed in all their patients. Since protein electrophoresis is not as sensitive as immunoelectrophoresis for the detection of M-protein, it is possible that the incidence of M-protein in EMP might have been higher in the earlier series if immunoelectrophoresis had been available. Local recurrence or disease dissemination was associated with the appearance, reappearance or elevation in the level of M-protein in both SPB and EMP, which suggests that M-protein is important for monitoring the disease course and change in the M-protein status following initial therapy should prompt reassessment and close follow-up.

Long-term local control for localised plasmacytomas can be achieved by adequate local treatment. Both SPB and EMP are sensitive to adequate doses of radiation therapy; however, there is no clear dose–response relationship. Most investigators recommend tumour doses for localised plasmacytomas ranging from 3500 to 5500 cGy (Kotner and Wang, 1972; Meyer and Schulz, 1974; Corwin and Lindberg, 1979; Woodruff *et al*., 1979*a*,*b*; Mill *et al*., 1980; Mendenhall *et al*., 1980; Harwood *et al*., 1981; Bush *et al*., 1981; Knowling *et al*., 1983; Greenberg *et al*., 1987; Frassica *et al*., 1989), whereas others have found that local failure can occur with doses greater than 6000 cGy in patients with EMP (Petrovich *et al*., 1977; Bush *et al*., 1981). Mendenhall *et al*. (1980), in a review of the literature, found a 94% local control rate for localised plasmacytomas with doses in excess of 4000 cGy compared with only 69% with doses less than 4000 cGy. Of our seven EMP patients treated with radiotherapy, none received less than 4500 cGy and local control was achieved in all. One patient developed cervical node recurrence just outside the margin of the previous radiation area, which supports the statement that regional lymph nodes should be included in the initial radiation fields for the treatment of EMP (Knowling *et al*., 1983; Mayr *et al*., 1990). In patients with SPB, especially in those with spinal lesions, we found that 3000 cGy in 3 weeks provided good local control with minimal morbidity, and radiation doses did not correlate with outcome.

The efficacy of chemotherapy for localised plasmacytomas is difficult to assess from the literature. Three recent reports demonstrated that adjuvant chemotherapy might increase the clearance rate of M-protein and could delay MM progression (Mayr *et al*., 1990; Jackson and Scarffe, 1990; Holland *et al*., 1992). However, we and Delauche-Cavallier *et al*. (1988) failed to find any benefit on the outcome of patients who received chemotherapy. Furthermore, an increased risk of therapy-related acute leukaemia following protracted courses of alkylating chemotherapy has been observed (Bergsagel *et al*., 1979; Delauche-Cavallier *et al*., 1988). Although the small number of patients receiving chemotherapy and lack of uniform treatment in the reported series, including this report, preclude any firm conclusion, we believe that adjuvant chemotherapy has limited value in the management of localised plasmacytomas if patients receive adequate local therapy with complete response. The role of adjuvant chemotherapy for patients with a persistent M-protein remains unsettled. Some authors claimed that persistence of an M-protein after local therapy indicated residual tumour or occult dissemination for which adjuvant therapy was suggested (Corwin and Lindberg, 1979; Woodruff *et al*., 1979*b*; Bataille and Sany, 1981; Jackson and Scarffe, 1990; Mayr *et al*., 1990); however, some of our patients with a persistent low level of M-protein have been in long-term apparent remission without further chemotherapy. Frassica *et al*. (1989) also found that a stable M-protein level might persist for a long time without therapy; they even demonstrated that persistence of M-protein after local therapy did not significantly influence outcome. In addition, the only two patients who developed MM in the current series received chemotherapy as the primary therapy. These observations suggest that the presence of a low constant level of M-protein after initial therapy, in the absence of other evidence of progression, does not necessarily indicate the need for adjuvant chemotherapy, and also chemotherapy does not prevent MM progression.

In the analysis of prognostic factors, there are conflicting results as to whether advanced age, presence of an M-protein or site of disease confers a poor prognosis. Our results are in agreement with those reported by Harwood *et al*. (1981), Chak *et al*. (1987), Frassica *et al*. (1989) and Jackson and Scarffe (1990) that M-protein does not significantly influence subsequent disease progression or survival, but are contrary to the results of others who found that the presence of M-protein indicated a higher incidence of MM progression and a worse survival (Knowling *et al*., 1983; Delauche-Cavallier *et al*., 1988; Dimopoulos *et al*., 1992; Holland *et al*., 1992). Bataille and Sany (1981) found that advanced age and spinal involvement were associated with a higher rate of progression, whereas we found that advanced age was unfavourable towards overall survival in SPB, but did not affect the relapse rate or disease-free survival in SPB or influence prognosis in EMP. Also, we failed to demonstrate that spinal lesions were associated with an increased risk of progression or a worse survival rate.

In three of the four patients who had a local relapse in the present series, this occurred within 3 years and in the remaining case at 9 years. Wiltshaw (1976), in an analysis of the combined series of localised plasmacytomas, found that local recurrence occurred mostly during the first 5 years after initial treatment but that relapse might occur more than 15 years later. Progression of SPB might take the form of metastasis to soft tissues or to other bones; similarly, EMP occasionally disseminated to the bones. Two of our patients with SPB ran a unique clinical course; they developed multiple sequential EMP and/or multiple new lytic bone lesions without bone marrow plasmacytosis. Bataille and Sany (1981) reported that new solitary lesions developed in 15% of their patients and, of these, 75% later converted to MM.

Whether the development of a secondary solitary plasmacytoma is a harbinger of ultimate conversion to MM and whether multiple bone metastases represents a particular pattern of spread different from MM are both unclear. Dimopoulos *et al.* (1992) observed a median time of 20 months for evolution from SPB to MM, with 68% of cases occurring within 3 years. Progression of SPB to MM was infrequent in our patients, as was the case for those of Delauche-Cavallier *et al.* (1988), compared with 40–75% of cases in other series (Meyer and Schulz, 1974; Woodruff *et al.*, 1979*b*; Bataille and Sany, 1981; Chak *et al.*, 1987; Frassica *et al.*, 1989; Dimopoulos *et al.*, 1992). The high incidence and rapid evolution to MM in the reported cases may be attributed to the understaging of the patients at initial investigation (Dimopoulos *et al.*, 1992).

Several reports in the literature have stressed that the difference between SPB and EMP is that EMP tends to remain localised whereas SPB appears to evolve more readily into MM. In the present study, the development into MM was seen in one patient each in both groups; the incidence and patterns of failure between SPB and EMP were not significantly different. The results of this present series contrast with the findings of a lower frequency of MM evolution from EMP than from SPB in other studies (Wiltshaw, 1976; Corwin and Lindberg, 1979; Knowling *et al.*, 1983; Greenberg *et al.*, 1987; Mayr *et al.*, 1990; Holland *et al.*, 1992). Our experience supports the observation of Meis *et al.* (1987) that SPB and EMP appear to be more closely related than has been previously recognised, and we also agree that it is useful to continue to classify and distinguish the two groups from each other for the purpose of treatment and continuing understanding of the natural course of localised plasmacytomas.

**Acknowledgements**
The authors thank Dr T Kuo for reviewing the pathological slides, Dr SK Lo for assistance in statistical analysis and Miss YF Wang for assistance in the preparation of the manuscript.

**References**

BATAILLE R AND SANY J. (1981). Solitary myeloma: clinical and prognostic features of a review of 114 cases. *Cancer*, **48**, 845–851.

BERGSAGEL DE, BAILEY AJ, LANGLEY GR, MACDONALD RN, WHITE DF AND MILLER AB. (1979). The chemotherapy of plasma-cell myeloma and the incidence of acute leukemia. *N. Engl. J. Med.*, **30**, 743–748.

BUSH SE, GOFFINET DR AND BAGSHAW MA. (1981). Extramedullary plasmacytoma of the head and neck. *Radiology*, **140**, 801–805.

CHAK LY, COX RS, BOSTWICK DG AND HOPPE RT. (1987). Solitary plasmacytoma of bone: treatment, progression, and survival. *J. Clin. Oncol.*, **5**, 1811–1815.

CORWIN J AND LINDBERG RD. (1979). Solitary plasmacytoma of bone vs. extramedullary plasmacytoma and their relationship to multiple myeloma. *Cancer*, **43**, 1007–1013.

DELAUCHE-CAVALLIER MC, LAREDO JD, WYBIER M, BARD M, MAZABRAUD A, DARNE LE BAIL JL, KUNTZ D AND RYCKEWAERT A. (1988). Solitary plasmacytoma of spine: long-term clinical course. *Cancer*, **61**, 1707–1714.

DIMOPOULOS MA, GOLDSTEIN J, FULLER L, DELASALLE K AND ALEXANIAN R. (1992). Curability of solitary bone plasmacytoma. *J. Clin. Oncol.*, **10**, 587–570.

ELLIS PA AND COLLS BM. (1992). Solitary plasmacytoma of bone: clinical features, treatment and survival. *Hematol. Oncol.*, **10**, 207–211.

FRASSICA DA, FRASSICA FJ, SCHRAY MF, SIM FH AND KYLE PA. (1989). Solitary plasmacytoma of bone: Mayo Clinic experience. *Int. J. Radiat. Oncol. Biol. Phys.*, **16**, 43–48.

GREENBERG P, PARKER RG, FU YS AND ABEMAYOR E. (1987). The treatment of solitary plasmacytoma of bone and extramedullary plasmacytoma. *Am. J. Clin. Oncol.*, **10**, 199–204.

HARWOOD AR, KNOWLING MA AND BERGSAGEL DE. (1981). Radiotherapy of extramedullary plasmacytoma of the head and neck. *Clin. Radiol.*, **32**, 31–36.

HOLLAND J, TRENKNER DA, WASSERMAN TH AND FINEBERG B. (1992). Plasmacytoma: treatment results and conversion to myeloma. *Cancer*, **69**, 1513–1517.

JACKSON A AND SCARFFE JH. (1990). Prognostic significance of osteopenia and immunoparesis at presentation in patients with solitary myeloma of bone. *Eur. J. Cancer*, **25**, 363–371.

KNOWLING MA, HARWOOD AR AND BERGSAGEL DE. (1983). Comparison of extramedullary plasmacytomas with solitary and multiple plasma cell tumors of bone. *J. Clin. Oncol.*, **1**, 255–262.

KOTNER LM AND WANG CC. (1972). Plasmacytoma of the upper air and food passages. *Cancer*, **30**, 414–418.

MAYR NA, WEN BC, HUSSEY DH, BURNS CP, STAPLES JJ, DOORNBOS JF AND VIGLIOTTI AP. (1990). The role of radiation therapy in the treatment of solitary plasmacytomas. *Radiother. Oncol.*, **17**, 293–303.

MEIS JM, BUTLER JJ, OSBORNE BM AND ORDONEZ NG. (1987). Solitary plasmacytomas of bone and extramedullary plasmacytomas. *Cancer*, **59**, 1475–1485.

MENDENHALL CM, THAR TL AND MILLION RR. (1980). Solitary plasmacytoma of bone and soft tissue. *Int. J. Radiat. Oncol. Biol. Phys.*, **6**, 1497–1501.

MEYER JE AND SCHULZ MD. (1974). Solitary myeloma of bone: a review of 12 cases. *Cancer*, **34**, 438–440.

MILL WB AND GRIFFITH R. (1980). The role of radiation therapy in the management of plasma cell tumors. *Cancer*, **45**, 647–652.

PAHOR AL. (1977). Extramedullary plasmacytoma of the head and neck, parotid and submandibular salivary glands. *J. Laryngol. Otol.*, **91**, 241–258.

PETROVICH Z, FISHKIN B, HITTLE RE, ACQUARELLI M AND BARTON R. (1977). Extramedullary plasmacytoma of the upper respiratory passages. *Int. J. Radiat. Oncol. Biol. Phys.*, **2**, 723–730.

TONG D, GRIFFIN TW, LARAMORE GE, KURTZ JM, RUSSELL AH, GROUDINE MT, HERRON T, BLASKO JC AND TESH DW. (1980). Solitary plasmacytoma of bone and soft tissues. *Radiology*, **135**, 195–198.

WILTSHAW E. (1976). The natural history of extramedullary plasmacytoma and its relation to solitary myeloma of bone and myelomatosis. *Medicine*, **55**, 217–238.

WOLLERSHEIM HCH, HOLDRINET RSG AND HAANEN C. (1984). Clinical course and survival in 16 patients with localized plasmacytoma. *Scand. J. Haematol.*, **32**, 423–428.

WOODRUFF RK, WHITTLE JM AND MALPAS JS. (1979*a*). Solitary plasmacytoma I: extramedullary soft tissue plasmacytoma. *Cancer*, **43**, 2340–2343.

WOODRUFF RK, MALPAS JS AND WHITE FE. (1979*b*). Solitary plasmacytoma II: solitary plasmacytoma of bone. *Cancer*, **43**, 2344–2347.